# Early Sterile Keratolysis Complication With Decellularized Porcine Corneal Inlay Implant: A Case Report and Cautionary Tale

**DOI:** 10.1097/ICO.0000000000003764

**Published:** 2024-11-26

**Authors:** Alfredo Borgia, Matteo Airaldi, Neil Lagali, Mohammad Ahmad, Amina Riaz, Stephen Kaye, Vito Romano

**Affiliations:** * St. Paul's Eye Unit, Department of Corneal Diseases, Royal Liverpool University Hospital, Liverpool, United Kingdom;; ‡ Department of Molecular and Translational Medicine, University of Brescia, Brescia, Italy;; § Department of Biomedical and Clinical Sciences, Faculty of Medicine, Linköping University, Linköping, Sweden;; ¶ Department of Ophthalmology, Sørlandet Hospital Arendal, Arendal, Norway;; † Department of Eye and Vision Science, Institute of Life Course and Medical Sciences, University of Liverpool, Liverpool, United Kingdom; and; ║ Ophthalmology Clinic, Department of Medical and Surgical Specialties, Radiological Sciences, and Public Health, University of Brescia, Italy.

**Keywords:** additive corneal surgery, keratoconus management, corneal melting, porcine corneal inlay

## Abstract

**Purpose:**

To describe an early sterile keratolysis associated with a decellularized porcine corneal inlay implant for keratoconus.

**Methods:**

This is a case report of a 23-year-old man with keratoconus who underwent lenticular intrastromal keratoplasty in his OD. Within 4 weeks, the patient presented with anterior sterile keratolysis and partial inlay extrusion, leading to surgical inlay removal.

**Results:**

After inlay removal, despite aggressive topical treatment including steroid drops, antibiotic ointment, and bandage contact lenses, the patient developed severe anterior scarring and corneal flattening, resulting in decreased visual acuity. Over a period of 4 months, the cornea underwent long-term remodeling, with vision improving to a best-corrected distance visual acuity of 0.1 logarithm of the minimum angle of resolution.

**Conclusions:**

The removal of the porcine corneal inlay because of severe keratolysis highlights the potential risks of this procedure. In addition, the spontaneous improvement in visual acuity over 4 months emphasizes the need for adequate healing time before further surgeries, such as keratoplasty.

## INTRODUCTION

Additive surgeries, including intracorneal lenticule inlays and ring segments, have emerged as promising interventions for enhancing corneal shape and visual outcomes in patients with keratoconus.^1,2^ However, despite their potential benefits, various complications have been reported after their clinical application, including infection, abnormal wound healing leading to corneal haze, and issues such as dislocation, decentration, and keratolysis, necessitating explantation in severe cases.^3–6^

In this case report, we describe an early sterile keratolysis after implantation of a porcine corneal inlay for keratoconus.

## CASE REPORT

A 23-year-old man diagnosed with grade 2 keratoconus, with a previous history of corneal cross-linking (Dresden protocol, epi-off) 3 years before, underwent lenticular intrastromal keratoplasty with the implantation of a decellularized porcine collagen lenticule (XENIA implants, Gebauer Medizintechnik GmbH, Cleveland, OH, USA - non-FDA approved) in his right eye at a different practice. Preoperatively, he presented with a maximum keratometry of 64.6 D and a thinnest corneal point of 464 μm.

The lenticules, derived from porcine corneas, undergo decellularization, intense cross-linking, sterilization, and packaging. These lenticules are transparent, disc-shaped, with a diameter of 7 mm and a thickness of 100 to 120 μm.

Based on the patient's records, a femtosecond laser (VisuMax, Carl Zeiss Meditec AG) was employed to create a 9-mm mid-stromal pocket at a depth of 140 μm from the corneal epithelium. The pocket dissection was performed using a SMILE procedure spatula. The Gebauer Corneal Lenticule was then inserted into the pocket with McPherson forceps and centered using the spatula.

Postoperative treatment included topical antibiotics (moxifloxacin 0.5% QDS for 7 days), topical steroids (dexamethasone 0.1% preservative-free QDS for 4 weeks), and cycloplegic drops (cyclopentolate 0.5% TDS for 3 days) in the operated eye.

**FIGURE 1.**
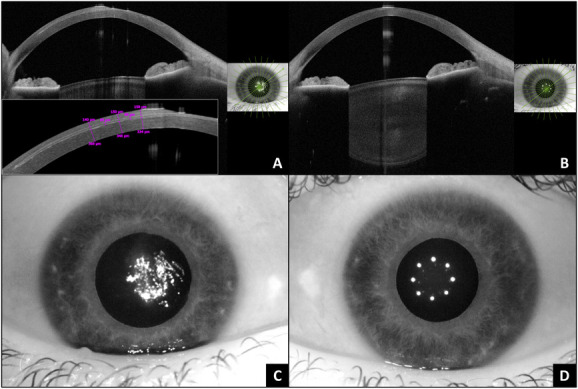
A, Anterior segment optical coherence tomography radial scan at 45 degrees displaying the area of keratolysis above the intracorneal lenticule implant (hyperreflective layer). Inlet: thicknesses of the cap and residual stromal bed. B, Anterior segment optical coherence tomography radial scan at 45 degrees illustrating the same area 10 months after the explantation of the intracorneal lenticule and complete reepithelialization. C, Infrared picture of the ocular surface showing an irregular corneal surface because of the exposure of the inlay implant in the paracentral superonasal cornea. D, Infrared picture of the ocular surface showing a more regular cornea without visible opacities at 10 months postoperatively.

Four weeks after surgery, he attended the corneal clinic of our tertiary referral hospital presenting with sterile keratolysis and a partial extrusion of the corneal inlay. The clinical examination revealed a large area of melted cornea centrally and paracentrally above the corneal inlay, measuring 5.4 mm vertically by 4.2 mm horizontally, showing the exposed inlay device (Fig. 1A).

Corneal sensation was absent in the area where the porcine implant was exposed and intact around the area of melting.

Cultures obtained showed no growth, and viral PCR for herpes virus was negative. In vivo confocal microscopy was not performed.

The medical history revealed no systemic disease to account for keratolysis, including history of herpes, autoimmune diseases, diabetes, and atopy.

Because of his condition, his uncorrected distance visual acuity declined from 0.9 to 1.4 logarithm of the minimum angle of resolution (LogMAR) in the OD.

Consequently, he required the removal of the inlay through surgical intervention.

**FIGURE 2.**
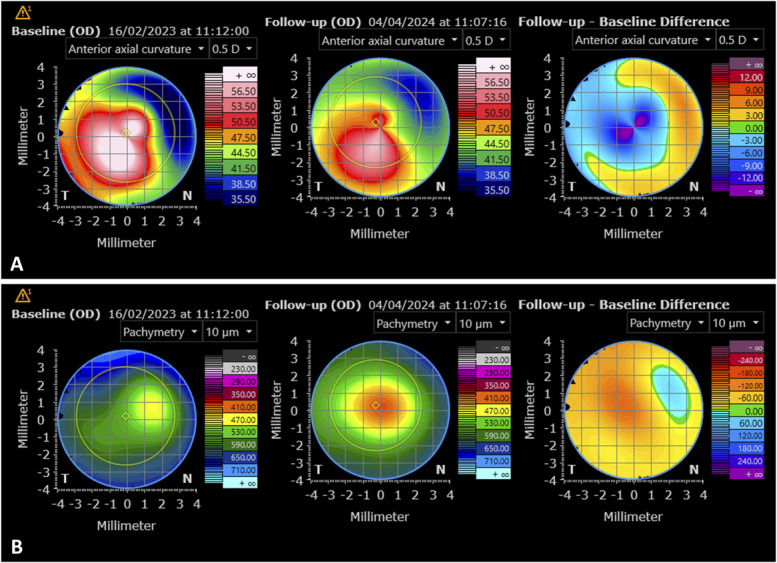
A, Anterior axial curvature topographic maps before the intracorneal porcine explantation and after the implant removal and the complete healing. A significant reduction in corneal steepening is evident. B, Pachymetry maps before the intracorneal porcine explantation, and after the implant removal (baseline) and after complete healing (follow-up). The baseline picture clearly shows an area of pachymetric thinning superonasally where the implant is completely exposed. In the follow-up picture, a more homogeneous pachymetry is present. Furthermore, the differential pachymetric map shows a diffuse reduction of pachymetry because of the porcine device explantation, along with a focal superonasal increase in pachymetry because of the remodeling and scarring process.

The surgical inlay removal was uneventful, and a bandage contact lens was applied at the end of the procedure and a postoperative treatment with steroid drops, and antibiotic ointment was started. A few weeks after inlay explantation, the patient developed severe anterior scarring and corneal flattening, resulting in decreased visual acuity. Over a 4-month period, the superficial corneal layer affected by keratolysis underwent long-term remodeling, transitioning from a scarred, concave surface to a mildly hazy area with regularized curvature and a reduction in the maximum curvature (from 65 to 59 D) (Fig. 2). After complete healing and corneal remodeling, his best-corrected visual acuity in the OD improved to 0.1 LogMAR (with a refraction of −0.50 sph −3.00 cyl @ 165 degrees), whereas before the operation, his best-corrected visual acuity was 0.8 LogMAR.

## DISCUSSION

For individuals with advanced keratoconus who cannot achieve satisfactory vision correction with glasses or tolerate contact lenses, deep anterior lamellar keratoplasty or penetrating keratoplasty have conventionally been the preferred surgical approaches to improve visual acuity.^7^

Nevertheless, corneal transplant surgery is associated with potential complications, such as graft rejection, graft failure, infection, glaucoma, astigmatism, corneal scarring, endothelial cell loss, and cataract development.^8^ Furthermore, PKs and DALKs may require general anesthesia, which may not be suitable for all patients. The need for postoperative care in the form of long-term immunomodulatory drops and the need for postoperative suture management can also constitute limiting factors to penetrating or lamellar keratoplasty. In contrast, femto-assisted additive corneal surgeries offer a less invasive alternative to corneal transplantation, potentially enhancing corneal shape and reducing high-order aberrations.^9^ However, despite being apparently less invasive compared with deep anterior lamellar keratoplasty or penetrating keratoplasty, and seemingly simple, additive corneal surgery is not without complications. Instances such as corneal melting, extrusion of intracorneal devices, microbial keratitis, or persistent epithelial defects may necessitate the removal of the intracorneal device and appropriate treatment with antibiotics, lubricating drops, and ointments.^3–6^

Previously, severe keratolysis has been documented after corneal additive surgery, in cases of intracorneal ring segments implantation and presbyopia inlay implantation.^5,6^

Intracorneal tissue implants were specifically devised to avoid PMMA-induced keratolysis, addressing the limitations associated with PMMA implants.^10,11^ In light of the potential to mitigate biocompatibility concerns, the adoption of allograft or potentially xenograft corneal material emerges as a possibly more advantageous option for corneal inlays, which have demonstrated relative initial clinical success.^2,9,10^ Rafat et al^12^ have recently demonstrated the safety and feasibility of a porcine bioengineered inlay implant, with visual improvements in all advanced keratoconic patients enrolled in the clinical trials, and no extrusion or dislocation of the implant.

The bioengineered implant is made in a laboratory from purified, medical-grade porcine collagen without contact to any cellular material, with the implant undergoing multiple sterilization procedures. By contrast, decellularized tissue previously contained multiple cell types before cellular removal and has an unknown extracellular composition and prior history.

To the best of our knowledge, this article represents the first documentation of decellularized porcine collagen corneal inlay extrusion. Our case report illustrates the successful resolution of keratolysis after removal of the implant, highlighting potential complications despite their perceived safety because of similarity of structure and composition to the human cornea. The severity of keratolysis necessitated inlay removal, underscoring the importance of vigilant long-term monitoring for optimal visual recovery.

There are multiple potential reasons for this occurrence. We excluded a possible postoperative infection because of history and appearance. A possible immune response to the implanted device or an incorrect placement of the device could also be responsible. The body may recognize foreign antigens or remnants of cellular material or DNA in the porcine tissue after decellularization and mount an immune reaction against them, evidence of which has been reported in prior studies using acellular porcine corneal stromal implantation.^11^ A further unknown factor in decellularization is whether the toxic compounds used in the decellularization process are completely removed from the implant, as even small amounts of residual chemicals could potentially trigger an immune response. The immune response after implantation can result in inflammation and subsequent keratolysis as the immune system attacks the corneal tissue. Poor surgical technique is also a factor that can contribute to keratolysis. Incorrect placement of the device, trauma during the surgical procedure, or improper handling of the corneal tissue can cause damage, leading to tissue breakdown. Mechanical stress exerted by the physical presence of the device on the corneal tissue can also contribute to keratolysis. This could be caused by mismatch of the stiffness of the decellularized cornea and the recipient cornea. This stress can cause wear and tear over time, leading to the breakdown of the tissue. Patients with preexisting corneal conditions are at a higher risk of developing keratolysis after implantation, as their corneas might already be compromised.

Finally, the visual improvement experienced by our patient after inlay explantation might be related to both flattening effect of the femtosecond laser cut and wound healing process at the 2 lamellar, host–implant interfaces, which can cause a contraction in the corneal stromal tissue. Given that the insertion and later removal of the implant on their own can lead to a significant net flattening of the cornea, these 2 effects might have contributed to a reduction in keratometry readings with improvement of visual acuity.

In conclusion, the necessity to remove the porcine corneal inlay because of severe keratolysis presented in this case report emphasizes the potential risks inherent in this seemingly innocuous additive procedure. Furthermore, the spontaneous improvement in visual acuity after inlay removal in our case underscores the need, whenever feasible, to await complete resolution of ocular inflammation and corneal remodeling before considering additional, invasive procedures such as corneal transplantation.
